# Supplementary figures and images for: Determination of progressive stages of type 2 diabetes in a 45% high-fat diet-fed C57BL/6J mouse model is achieved by utilizing both fasting blood glucose levels and a 2-hour oral glucose tolerance test
Source: PLoS One. 2023 Nov 14;18(11):e0293888. doi: 10.1371/journal.pone.0293888 (PMC10645328; doi:10.1371/journal.pone.0293888)

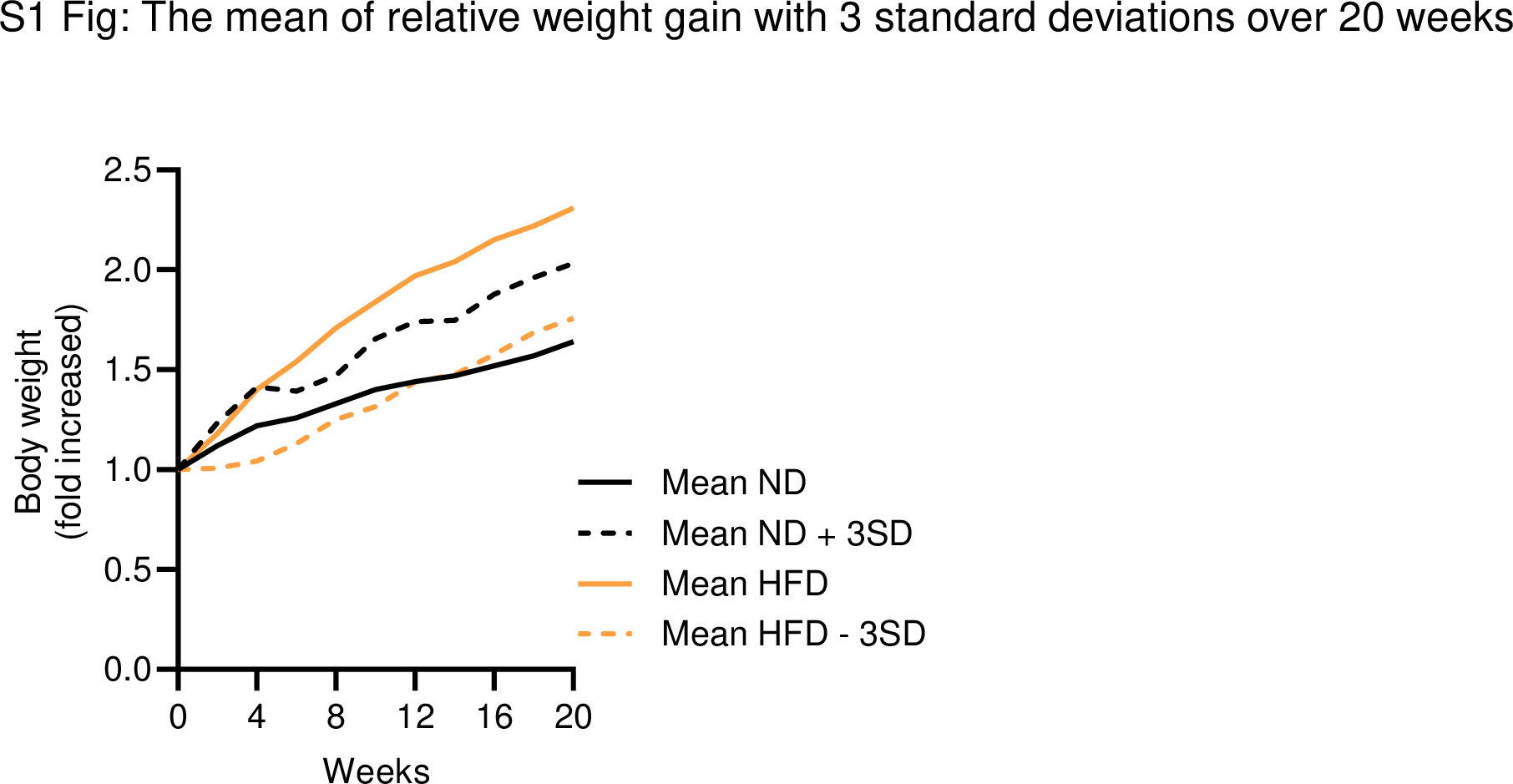

Supplement: S1 Fig — Mean relative weight gain of ND group (black, solid line) with their upper 3 standard deviations (black, dash line). Mean relative weight gain of HFD group (orange, solid line) with their lower 3 standard deviations (orange, dash line). (TIF) [file pone.0293888.s001.tif]

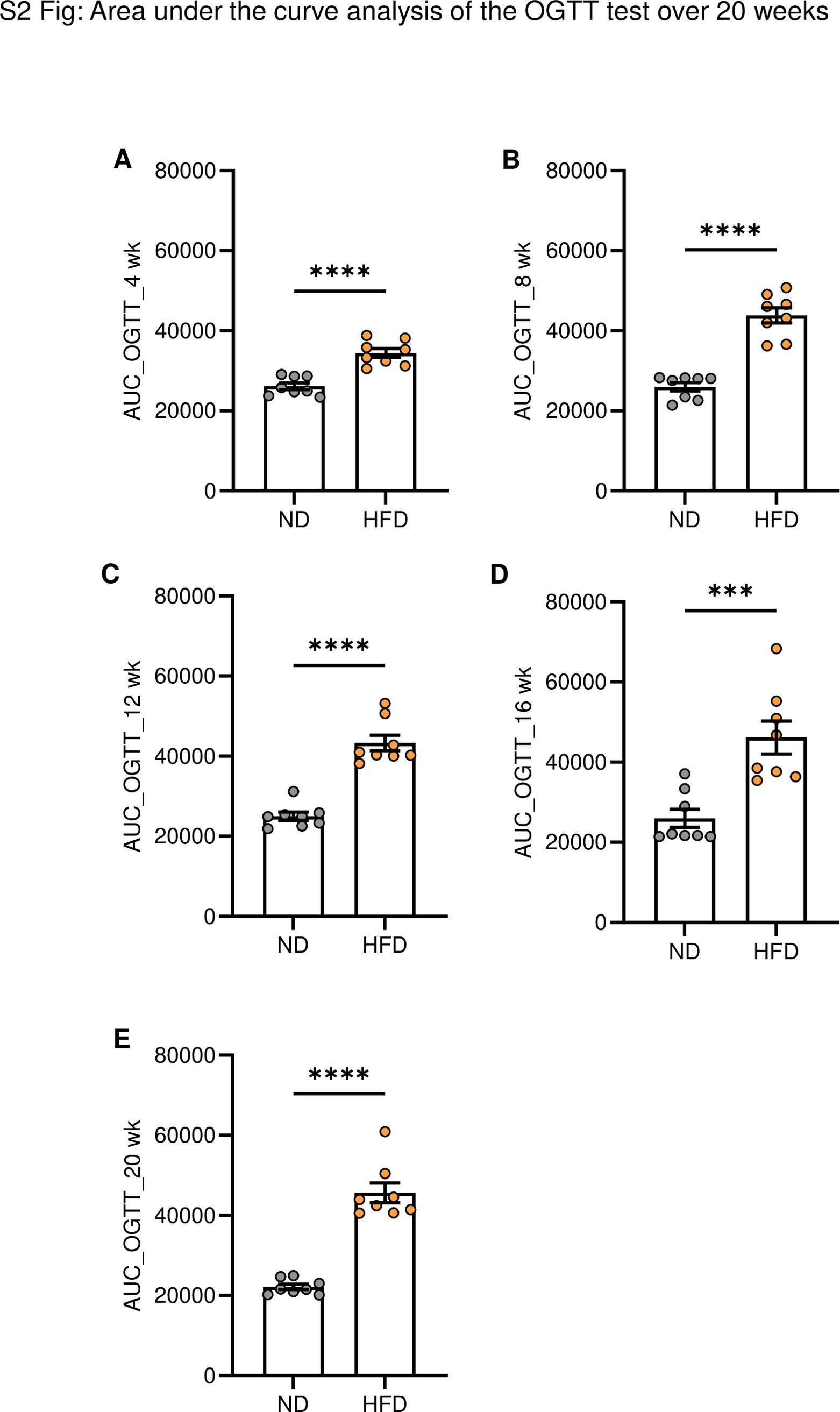

Supplement: S2 Fig — OGTT test were compared between ND group and HFD group at (A) week four, (B) week eight, (C) week twelve, (D) week sixteen, and (E) week twenty by area under the curve analysis. Data represent mean ± SEM. *** p < 0.001, **** p < 0.0001 by two-tailed student’s t-test. (TIF) [file pone.0293888.s002.tif]

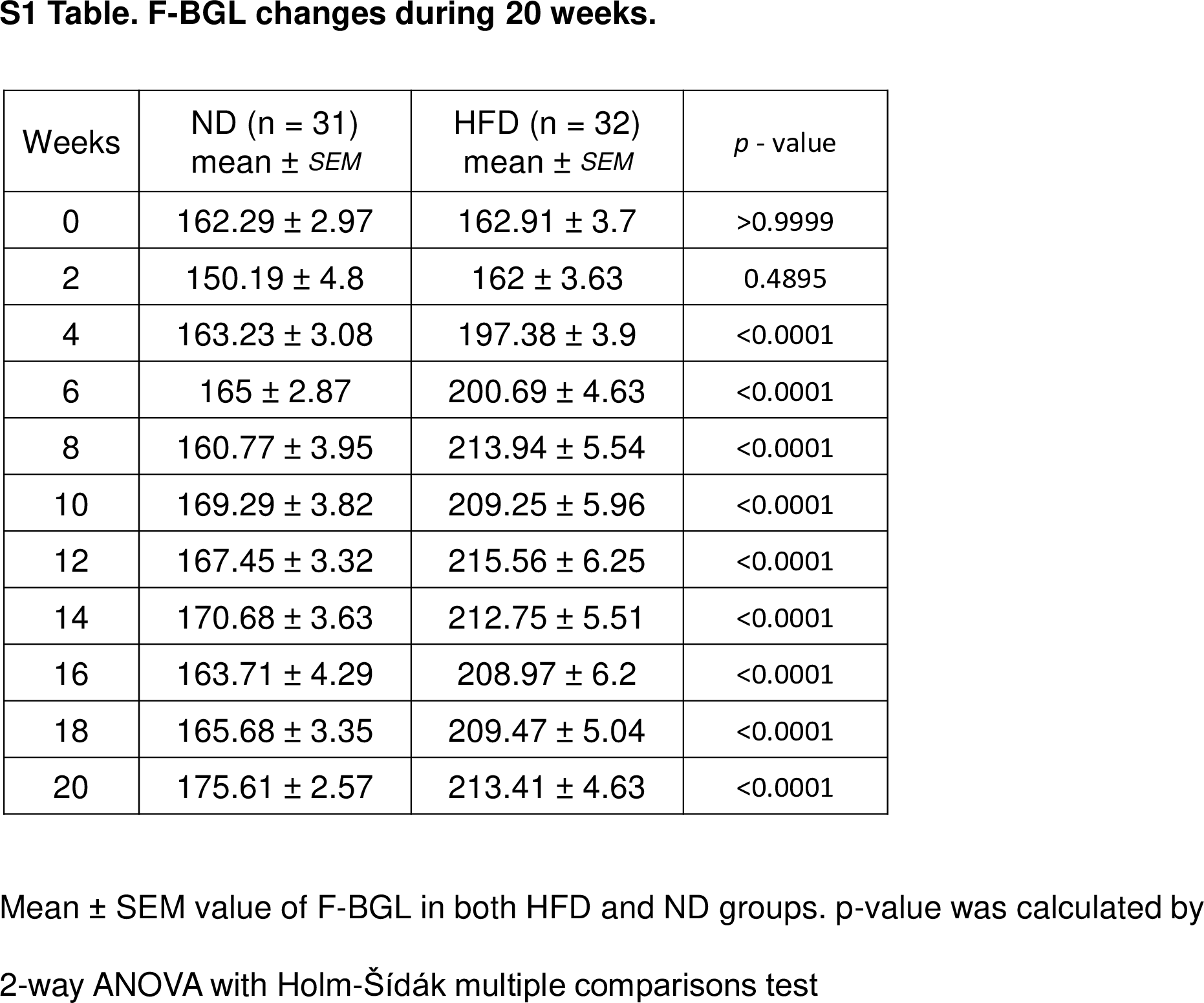

Supplement: S1 Table — Mean ± SEM value of F-BGL in both HFD and ND groups. p-value was calculated by 2-way ANOVA with Holm-Šídák multiple comparisons test. (TIF) [file pone.0293888.s003.tif]
