# Supplementary material for: Determination of progressive stages of type 2 diabetes in a 45% high-fat diet-fed C57BL/6J mouse model is achieved by utilizing both fasting blood glucose levels and a 2-hour oral glucose tolerance test
Source: PLoS One. 2023 Nov 14;18(11):e0293888. doi: 10.1371/journal.pone.0293888 (PMC10645328; doi:10.1371/journal.pone.0293888)
Supplement: S1 File — (PDF) [file pone.0293888.s004.pdf]

Fig 1A: Body weight

Weeks	ND (n = 31) mean ± SEM	HFD (n = 32) mean ± SEM
0	18.66 ± 0.19	18.78 ± 0.21
2	20.86 ± 0.25	22.19 ± 0.3
4	22.71 ± 0.32	26.21 ± 0.47
6	23.54 ± 0.28	28.9 ± 0.56
8	24.74 ± 0.3	32.1 ± 0.57
10	26.23 ± 0.31	34.48 ± 0.7
12	27.1 ± 0.36	37.03 ± 0.68
14	27.6 ± 0.32	38.28 ± 0.71
16	28.66 ± 0.41	40.3 ± 0.66
18	29.48 ± 0.41	41.72 ± 0.65
20	30.76 ± 0.42	43.27 ± 0.6

Factors: “Body weight” and “Time”

Subjects: ND (n = 31), HFD (n = 32)

$F(1, 61) = 164.5$, $p < 0.001$

Missing values: 0

Fig 1B: Cholesterol

Weeks	ND (n = 8) mean ± SEM	HFD (n = 8) mean ± SEM
4	70.81 ± 3.18	111.94 ± 6.7
12	66.25 ± 1.72	131.19 ± 5.27
16	68.35 ± 1.85	169.51 ± 12.91
20	81.24 ± 3.7	206.82 ± 21.83

Factors: “Cholesterol” and “Time”

Subjects: ND (n = 8), HFD (n = 8)

$F(1, 14) = 133.2$, $p < 0.001$

Missing values: 0

Fig 1C: Triglyceride

Weeks	ND (n = 8) mean ± SEM	HFD (n = 8) mean ± SEM
4	19.06 ± 3.23	8.06 ± 1.31
12	39.5 ± 2.92	21.61 ± 2.06
16	27.68 ± 0.95	22.65 ± 2.59
20	88.2 ± 7.6	44.44 ± 7.21

Factors: “Triglyceride” and “Time”

Subjects: ND (n = 8), HFD (n = 8)

$F(1, 14) = 50.71$, $p < 0.001$

Missing values: 0

Fig 1D: C-peptide

Weeks	ND (n = 8) mean ± SEM	HFD (n = 8) mean ± SEM
4	0.78 ± 0.06	1.52 ± 0.11
8	0.77 ± 0.14	2.61 ± 0.39
12	0.89 ± 0.14	1.93 ± 0.28
20*	0.98 ± 0.22	2.63 ± 0.33

**Week 20: ND (n = 7); HFD (n = 6)*

Factors: “C-peptide” and “Time”

Subjects: ND (n = 8), HFD (n = 8)

$F(1, 14) = 62.31$, $p < 0.001$

Missing values: 3 (1 value from ND group week 20; 2 values from HFD group week 20)

Fig 1E: Calorie intake

Weeks	ND (n = 8) mean ± SEM	HFD (n = 8) mean ± SEM
1	7.63 ± 0.25	10.86 ± 0.17
2	7.84 ± 0.22	9.57 ± 0.32
3	7.77 ± 0.22	10.08 ± 0.26
4	7.67 ± 0.24	10.49 ± 0.28
5	7.96 ± 0.25	10.04 ± 0.27
6	7.95 ± 0.23	10.25 ± 0.37
7	7.82 ± 0.25	10.89 ± 0.59
8	7.7 ± 0.24	10.27 ± 0.36
9	7.97 ± 0.29	9.49 ± 0.23
10	8.22 ± 0.36	9.61 ± 0.29
11	7.94 ± 0.23	10.05 ± 0.33
12	7.91 ± 0.21	10.17 ± 0.35
13	7.2 ± 0.3	9.15 ± 0.36
14	8.05 ± 0.27	9.77 ± 0.2
15	8.13 ± 0.27	9.65 ± 0.2
16	7.85 ± 0.24	9.68 ± 0.24
17	8.06 ± 0.3	9.63 ± 0.23
18	8.25 ± 0.25	9.71 ± 0.24
19	7.99 ± 0.27	9.9 ± 0.19
20	7.84 ± 0.21	9.61 ± 0.28

Factors: “Calorie intake” and “Time”

Subjects: ND (n = 8), HFD (n = 8)

$F(1, 14) = 73.34$, $p < 0.001$

Missing values: 0

Fig 1F: Water intake

Weeks	ND (n = 8) mean ± SEM	HFD (n = 8) mean ± SEM
1	4 ± 0.22	2.7 ± 0.08
2	4.01 ± 0.2	2.75 ± 0.11
3	3.7 ± 0.19	2.64 ± 0.19
4	3.86 ± 0.23	2.68 ± 0.09
5	4.05 ± 0.33	2.69 ± 0.18
6	3.97 ± 0.13	2.54 ± 0.11
7	3.67 ± 0.18	2.38 ± 0.11
8	4.03 ± 0.2	2.57 ± 0.11
9	3.67 ± 0.17	2.38 ± 0.1
10	3.94 ± 0.24	2.57 ± 0.07
11	3.73 ± 0.15	2.34 ± 0.06
12	4 ± 0.21	2.42 ± 0.08
13	3.53 ± 0.23	2.27 ± 0.07
14	3.86 ± 0.14	2.35 ± 0.07
15	3.61 ± 0.23	2.07 ± 0.07
16	4.08 ± 0.26	2.42 ± 0.07
17	3.81 ± 0.21	2.16 ± 0.08
18	3.97 ± 0.21	2.31 ± 0.12
19	3.72 ± 0.2	2.07 ± 0.06
20	3.86 ± 0.18	2.55 ± 0.19

Factors: “water intake” and “Time”

Subjects: ND (n = 8), HFD (n = 8)

$F(1, 14) = 103.2$, $p < 0.001$

Missing values: 0

Fig 2A: F-BGL

Weeks	ND (n = 31)<br>mean ± SEM	HFD (n = 32)<br>mean ± SEM
0	162.29 ± 2.97	162.91 ± 3.7
2	150.19 ± 4.8	162 ± 3.63
4	163.23 ± 3.08	197.38 ± 3.9
6	165 ± 2.87	200.69 ± 4.63
8	160.77 ± 3.95	213.94 ± 5.54
10	169.29 ± 3.82	209.25 ± 5.96
12	167.45 ± 3.32	215.56 ± 6.25
14	170.68 ± 3.63	212.75 ± 5.51
16	163.71 ± 4.29	208.97 ± 6.2
18	165.68 ± 3.35	209.47 ± 5.04
20	175.61 ± 2.57	213.41 ± 4.63

Factors: “F-BGL” and “Time”

Subjects: ND (n = 31), HFD (n = 32)

$F(1, 61) = 152.6$, $p < 0.001$

Missing values: 0

Fig 2G: 2h-OGTT

Weeks	ND (n = 8)<br>mean ± SEM	HFD (n = 8)<br>mean ± SEM
4	162.38 ± 2.18	222.25 ± 13.13
8	163.13 ± 6.21	265.38 ± 18.6
12	162.88 ± 9.29	309.88 ± 17.71
16	172.25 ± 12.1	378.13 ± 38.93
20	144.63 ± 8.94	378.63 ± 43.03

Factors: “2h-OGTT” and “Time”

Subjects: ND (n = 8), HFD (n = 8)

$F(1, 14) = 71.38$, $p < 0.001$

Missing values: 0
