# Supplementary material for: Determination of progressive stages of type 2 diabetes in a 45% high-fat diet-fed C57BL/6J mouse model is achieved by utilizing both fasting blood glucose levels and a 2-hour oral glucose tolerance test
Source: PLoS One. 2023 Nov 14;18(11):e0293888. doi: 10.1371/journal.pone.0293888 (PMC10645328; doi:10.1371/journal.pone.0293888)
Supplement: Attachment — Submitted filename: Response to reviewers.docx [file pone.0293888.s006.docx]

October 18^th^, 2023

Dear Dr. Peng Gao

PLOS ONE Academic Editor,

We really appreciate to re-submit our manuscript entitled "***Determination of progressive stages of type 2 diabetes in a 45% high-fat diet-fed C57BL/6J mouse model is achieved by utilizing both fasting blood glucose levels and a 2-hour oral glucose tolerance test***" [PONE-D-23-24628R]. We are grateful to the reviewers and editors for their helpful and supportive comments. We have addressed the issue raised by the editor in the response letter and the revised manuscript with the track changes. Here is a point-by-point response to the editor’s comments.

**Journal Requirements:**

1. Please review your reference list to ensure that it is complete and correct. If you have cited papers that have been retracted, please include the rationale for doing so in the manuscript text, or remove these references and replace them with relevant current references. Any changes to the reference list should be mentioned in the rebuttal letter that accompanies your revised manuscript. If you need to cite a retracted article, indicate the article’s retracted status in the References list and also include a citation and full reference for the retraction notice.

Thank you for the comment. We have checked all the references that were cited in this manuscript. As of now, none of them is a retracted paper.

**Additional Editor Comments:**

There are two remaining issues.

1) The authors have included their F-values and degrees of freedom in their response to reviewers, but have not embedded them within the manuscript.

Thank you for the comment. We added F-values and degrees of freedom in the S1 File at the Supporting information section in the revised manuscript, line 331-332.

2) The availability of raw data should be declared in the methods section.

Thank you for the comment. We removed the “Data availability” in the methods section, lines 118 – 120. Raw data was provided in the S1 File in the Supporting information section in the revised manuscript, lines 331-332.

[Note: HTML markup is below. Please do not edit.]

Reviewers' comments:

Reviewer's Responses to Questions

Comments to the Author

1. If the authors have adequately addressed your comments raised in a previous round of review and you feel that this manuscript is now acceptable for publication, you may indicate that here to bypass the “Comments to the Author” section, enter your conflict of interest statement in the “Confidential to Editor” section, and submit your "Accept" recommendation.

Reviewer #1: All comments have been addressed

Thank you for your comment.

Reviewer #2: (No Response)

2. Is the manuscript technically sound, and do the data support the conclusions?

The manuscript must describe a technically sound piece of scientific research with data that supports the conclusions. Experiments must have been conducted rigorously, with appropriate controls, replication, and sample sizes. The conclusions must be drawn appropriately based on the data presented.

Reviewer #1: Yes

Reviewer #2: Yes

3. Has the statistical analysis been performed appropriately and rigorously?

Reviewer #1: Yes

Reviewer #2: Yes

4. Have the authors made all data underlying the findings in their manuscript fully available?

The PLOS Data policy requires authors to make all data underlying the findings described in their manuscript fully available without restriction, with rare exception (please refer to the Data Availability Statement in the manuscript PDF file). The data should be provided as part of the manuscript or its supporting information, or deposited to a public repository. For example, in addition to summary statistics, the data points behind means, medians and variance measures should be available. If there are restrictions on publicly sharing data—e.g. participant privacy or use of data from a third party—those must be specified.

Reviewer #1: Yes

Reviewer #2: No

5. Is the manuscript presented in an intelligible fashion and written in standard English?

PLOS ONE does not copyedit accepted manuscripts, so the language in submitted articles must be clear, correct, and unambiguous. Any typographical or grammatical errors should be corrected at revision, so please note any specific errors here.

Reviewer #1: Yes

Reviewer #2: Yes

6. Review Comments to the Author

Please use the space provided to explain your answers to the questions above. You may also include additional comments for the author, including concerns about dual publication, research ethics, or publication ethics. (Please upload your review as an attachment if it exceeds 20,000 characters)

Reviewer #1: This study sought to determination of progressive stages of type 2 diabetes using a mouse model. Thank you for addressing my questions. I do not have further comments.

Thank you for your comment.

Reviewer #2: (No Response)

7. PLOS authors have the option to publish the peer review history of their article (what does this mean?). If published, this will include your full peer review and any attached files.

If you choose “no”, your identity will remain anonymous but your review may still be made public.

Do you want your identity to be public for this peer review? For information about this choice, including consent withdrawal, please see our Privacy Policy.

Reviewer #1: No

Reviewer #2: No

We hope that you will find the revised manuscript satisfactory for publication. We look forward to hearing from you in due time regarding our submission and to respond to any further questions and comments you may have.

Sincerely,

**Chung-Gyu Park**, MD, PhD

Professor, Seoul National University College of Medicine

103 Daehak-ro Jongno-gu, Seoul 03080, Korea

Tel. +82-2-740-8007, 8308 Fax +82-2-743-0881

E-mail: chgpark@snu.ac.kr, chgpark@gmail.com
